# Lipid Metabolism in Metabolic-Associated Steatotic Liver Disease (MASLD)

**DOI:** 10.3390/metabo14010012

**Published:** 2023-12-23

**Authors:** Majid Mufaqam Syed-Abdul

**Affiliations:** Toronto General Hospital Research Institute, University Health Network, University of Toronto, Toronto, ON M5G 1L7, Canada; majidmufaqam.syedabdul@uhn.ca

**Keywords:** MASLD, MASH, cholesterol, DNL, fatty acid oxidation

## Abstract

Metabolic-associated steatotic liver disease (MASLD) is a cluster of pathological conditions primarily developed due to the accumulation of ectopic fat in the hepatocytes. During the severe form of the disease, i.e., metabolic-associated steatohepatitis (MASH), accumulated lipids promote lipotoxicity, resulting in cellular inflammation, oxidative stress, and hepatocellular ballooning. If left untreated, the advanced form of the disease progresses to fibrosis of the tissue, resulting in irreversible hepatic cirrhosis or the development of hepatocellular carcinoma. Although numerous mechanisms have been identified as significant contributors to the development and advancement of MASLD, altered lipid metabolism continues to stand out as a major factor contributing to the disease. This paper briefly discusses the dysregulation in lipid metabolism during various stages of MASLD.

## 1. Metabolic-Associated Steatotic Liver Disease

MASLD is a recent nomenclature that has been approved to broaden the diagnostic criteria and to avoid stigmatization for a previously known condition called nonalcoholic fatty liver disease (NAFLD) [1], which encompasses a spectrum of liver conditions, ranging from simple fatty liver (hepatic steatosis), which can be detected through imaging or histological methods, to MASH, which involves inflammation and can lead to more severe liver damage [2]. Currently, characteristics indicative of MASLD have been observed in nearly a quarter of the global population [3,4], with these prevalence rates showing an upward trend [5]. This escalating occurrence of MASLD closely mirrors the concurrent rise in obesity and constitutes a significant contributing factor to the expanding burden of chronic hepatic diseases on a worldwide scale [3,4,5,6,7,8,9,10]. As anticipated, in concurrence with the rising prevalence of MASLD, there has been a significant 2.0–2.5-fold increase in the incidence of MASH in recent years [4,11] which was robustly linked to liver-related morbidity [12,13]. Additionally, it is important to highlight that projections indicate that MASH is nearing the position of becoming the second most prevalent causative factor necessitating liver transplantation [14].

## 2. Pathophysiological Changes at the Molecular Level in MASLD

Serological studies and numerous genomic studies performed on hepatocytes sourced from patients diagnosed with MASLD and individuals undergoing bariatric surgery consistently reveal a noticeable increase in several biochemical parameters [15] and the upregulation of key enzymes integral to the de novo lipogenesis (DNL) pathway [16,17,18,19,20,21]. As a master regulator of the DNL pathway, sterol regulatory element-binding protein 1c (SREBP1c), primarily activated by insulin, exhibited a significant increase in MASLD patients compared to those without MASLD [17,20,22,23,24,25], underscoring its central role in governing this metabolic process. Contrary to these findings, single-cell RNA sequencing (scRNA-seq) combined with computational network analyses to explore lipid signatures in mice with MASLD showed that, despite its traditional role as a driver of lipid synthesis, high SREBP1 expression is not predictive of hepatic lipid accumulation in non-alcoholic fatty liver disease (NAFLD); instead, the study identifies the constitutive androstane receptor (CAR) as a key player in regulating functional modules associated with cholesterol homeostasis, bile acid metabolism, fatty acid metabolism, and estrogen response, demonstrating its correlation with steatohepatitis in human livers [26]. Notably, among the subsequent enzymes regulated by SREBP1c, the isoforms of acetyl-CoA carboxylase (ACC) exhibited a remarkable increase, i.e., a more than eight-fold increase in expression in MASLD patients compared to those with normal liver profiles [16,17,19]. Similarly, hepatic fatty acid (FA) synthase (FASN) gene expression was significantly higher in MASLD patients i.e., one- to five-fold higher compared to healthy subjects [16,17,18,20]. Lastly, the expression of the stearoyl-CoA desaturase (SCD-1) gene exhibited an approximate nine-fold increase in individuals diagnosed with MASLD [16].

In addition to an increase in DNL enzyme expression, patients with MASLD also exhibited altered expression of FA binding protein (FABP), FA transport protein (FATP) [16,17,21,27,28], and CD36 [16,21,28]—genes responsible for FA uptake. Moreover, genes associated with triacylglycerols (TAGs) synthesis, including diacylglycerol o-acyltransferase 2 (DGAT2) [19] and microsomal triglyceride transfer protein (MTTP) [16,17,29], along with genes impacting very-low-density lipoprotein (VLDL) kinetics, exemplified by apolipoprotein B100 (apoB100) [16,17,29], exhibited increased expression levels.

Furthermore, genes related to the oxidation of FAs, including peroxisome proliferator-activated receptor gamma (PPAR-γ) [27] and carnitine palmitoyltransferase 1 (CPT1) [16,27], were upregulated, while PPAR-γ coactivator 1α (PGC-1α) was downregulated [30]. It is worth noting that Moore et al. reported decreased expression of FA oxidation genes in MASLD patients [31]. MASH patients, in comparison with MASLD patients, exhibited lower expression of peroxisome proliferator-activated receptor α (PPAR-α), MTTP, and apoB100, but no changes were observed in SREBP1c, FASN, DGAT1 and 2, FABP and FATP, and CD36 [32,33,34]. In the human liver, we have recently shown a significant positive relationship between the protein expression of FASN and DNL measured isotopically [35,36]. Lastly, cirrhotic patients and hepatocellular carcinoma (HCC) patients exhibited increased CD36 expression and reduced PPAR-α expression [33]. It is important to note that the majority of these studies utilized gene/protein expression as a read-out, which, like every other method, has some limitations, including the overlooking of post-transcriptional and post-translational regulation, temporal dynamics, functional redundancy, cellular heterogeneity, spatial variation, and the necessity to consider functional connectivity and interactions within metabolic networks.

## 3. Lipid Synthesis in MASLD

### 3.1. Fatty Acids

**Figure 1:**
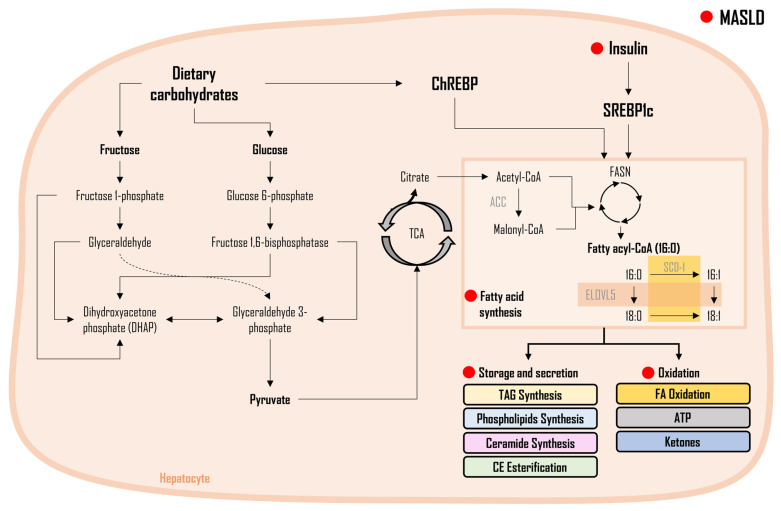
Fatty acid synthesis and regulation in the development of MASLD. Legend: This figure illustrates the intricate process of Fatty Acid (FA) synthesis and its regulation. The synthesis of FAs involves a complex biochemical pathway, primarily driven by the activities of key enzymes such as ACC; FASN; SCD-1; and elongases (ELOV). The master regulator SREBP1c, influenced by insulin levels, governs the activation of enzymes in de novo lipogenesis (DNL). Additionally, ChREBP serves as a regulator activated during postprandial states and hyperglycemia, impacting gene transcription and enzymatic activity in the DNL pathway. The red dot (●) represents changes altered during MASLD pathogenesis. Figure abbreviations: ACC—acetyl-CoA carboxylase; CE—cholesterol ester; ELOVL5—elongases 5; FASN—fatty acid synthase; SCD-1—stearoyl-CoA desaturase-1; TCA—tricarboxylic cycle; and TAG—triacylglycerols.

As shown in Figure 1, FA synthesis is a complex biochemical process responsible for the synthesis of FAs, utilizing glycerol and carbon molecules. When FAs are generated from non-lipid sources, notably carbohydrates, this metabolic pathway is termed DNL [37,38]. Serving as the master regulator, SREBP1c controls the activation of enzymes of the DNL pathway, collectively governing the complex process of FA synthesis at the molecular level through their specific functions. The activation of SREBP-1c has been demonstrated to be regulated by insulin concentrations, with higher insulin levels leading to increased SREBP-1c activation [22]. At the molecular level, crucial enzymes involved in FA synthesis include ACC, which catalyzes acetyl-CoA carboxylation, a pivotal step in FA synthesis. FASN plays an orchestrating role in the intricate assembly of FAs, ensuring the formation of these essential molecules. SCD-1 is responsible for facilitating desaturation reactions, crucial for modifying FA chains to confer specific properties. Additionally, a group of enzymes known as elongases (ELOV) [39] contributes significantly by elongating FA chains, collectively ensuring the production of various FA species. These newly-made FAs can then be used either as a source of energy (i.e., for the synthesis of adenosine triphosphate, ATP), for the synthesis of TAGs or phospholipids (PLs), or for the esterification of free cholesterols to form cholesterol esters (CE) [40].

The onset of MASLD is perhaps driven by insulin resistance, triggered by overnutrition, caloric surplus, or physiological changes [41,42,43,44,45]. This condition leads to hyperinsulinemia, which in turn results in an elevation in SREBP1c expression and the consequential upregulation of essential enzymes within the pathway of DNL (ACC, FASN, SCD-1). These observations were consistent in animal models as well as in in vitro studies, providing valuable insights into the mechanisms underlying MASLD [46,47,48,49,50,51,52,53,54,55,56]. Carbohydrate response element-binding protein (ChREBP) serves as another regulator of hepatic DNL. It is primarily activated during a postprandial state and hyperglycemia [57,58,59] and has been reported to increase gene transcription or directly enhance enzymatic activity [58,60,61,62]. In contrast, ChREBP downregulation in mice liver, known for its primary role in maintaining glucose and lipid homeostasis [63,64,65,66], significantly mitigated steatosis induced by high-carbohydrate feeding. Furthermore, with ChREBP downregulation, endogenous glucose production increased, FA oxidation decreased, and insulin resistance increased [67]. Intriguingly, the insulin resistance state reversed in a mouse model of diabetes and insulin resistance (i.e., *ob*/*ob* mice) when ChREBP was downregulated [58,68]. Although inhibiting ChREBP decreases DNL caused by fructose and elevates TAG levels [69], it has been linked to adverse effects such as fructose intolerance, gastrointestinal distress including diarrhea and irritable bowel syndrome, as well as cholesterol-induced liver damage [69,70,71,72]. When adenoviral overexpression of ChREBP is induced in mice, intrahepatic triglyceride (IHTG) levels increased, the degree of steatosis advanced, but insulin action and glucose tolerance improved [59]. In individuals with obesity, a direct link has been noticed between hepatic mRNA expression of ChREBPβ and the presence of insulin resistance and steatosis. Among individuals with MASH, an inverse association has been documented between ChREBPβ levels and insulin resistance [58,59,73]. Although the exact causes for these disparities among studies are ambiguous, it has been suggested that variations could be attributed to differences in dietary, environmental, or genetic factors [58]. In brief, DNL seems to be triggered by mechanisms that encompass both insulin-dependent as well as insulin-independent pathways, which entail the participation of SREBP1c- and ChREBP-signaling pathways [74]. This process contributes to approximately 26% of the overall FA content within the liver’s lipid pool [75]. Early investigations have revealed that elevated DNL is an initial occurrence that differentiates individuals with MASLD from those who are equally obese but exhibit low IHTG levels [75,76,77,78]. Subsequent studies have corroborated these results, demonstrating that enhanced DNL is evident in fatty liver patients compared to those within the healthy or normal range [22,38,79,80,81,82]. Consistent with these results, in our recent findings, liver-DNL was significantly different between healthy controls and individuals whose liver histology exhibited one or more characteristics of MASLD (i.e., steatosis, inflammation, and/or ballooning); however, in individuals whose liver histology showed the presence of fibrosis (in addition to the aforementioned characteristics), liver DNL was significantly lower (similar to the level of healthy individuals). These findings were likely because there is no synthetic machinery to generate FAs when the tissue becomes fibrotic [35]. Despite the increasing prevalence of MASLD worldwide over the past two decades [3,4,83], there are currently no approved pharmaceutical treatments available for MASLD. Recognizing the significant role of DNL in MASLD pathogenesis [57,76,78,84,85], pharmaceutical industry researchers have been diligently engaged in the design and exploration of drug candidates [86,87,88,89] aimed at inhibiting the key enzymes of the DNL pathway [46,90,91,92,93,94,95]. Several DNL inhibitors have exhibited their capacity to decrease IHTG levels in both MASLD individuals and those exhibiting features of metabolic syndrome—a cluster of characteristics that increases the risk of developing MASLD [54,90,91,92,93,95,96].

#### Saturated vs. Unsaturated FAs in MASLD

Both the mono-(MUFA) and poly-unsaturated FAs (PUFA) have been studied briefly in relation to MASLD pathophysiology. Observational studies reported a significant relationship between low PUFA concentrations and MASLD presence. As reviewed by Yan et al., in a meta-analysis of 18 studies involving 1424 patients, PUFA supplementation (omega-3 in particular) resulted in lower hepatic enzyme levels and fat accumulation compared to control groups [97]. Similarly, impaired expression and activity of FADS1, a key desaturase in lipid metabolism, has been shown to contribute to altered FA profiles in MASH, leading to an imbalance in the n-6:n-3 ratio and impacting the synthesis of pro-inflammatory lipid-signaling molecules [98]. Others have also utilized the n-6:n-3 ratio of long-chain PUFA which is an indication of increased synthesis and decreased oxidation/secretion of FAs, and reported a relationship between the n-6/n3 ratio and steatosis [99]. Additionally, long-chain PUFA depletion may arise from impaired desaturation of PUFA, linked to insufficient intake of precursors like 18:3, n-3, increased 18:1, n-9 trans isomer intake causing desaturase inhibition, and elevated long-chain PUFA peroxidation under oxidative stress [99]. Indeed, in a lipidomic analysis, a significantly higher n-6:n-3 ratio was observed in MASH patients compared to individuals with MASLD or those who were in a control group [100]. Additionally, Puri et al. also reported significantly lower long-chain PUFA concentrations in MASH patients compared to the control group [100]. In research undertaken by Zhu et al., an analysis of the 2017–2018 National Health and Nutrition Examination Survey data revealed a negative correlation between the ratio of unsaturated to saturated FAs and the likelihood of experiencing significant liver fibrosis in the U.S. population [101]. Specifically, higher intakes of dietary PUFA and linoleic acid were linked to a decreased risk of significant liver fibrosis. In an observational clinical trial [102], a multivariable logistic regression analysis adjusted for BMI, age, and PNPLA3 (I148M) genotype revealed a significant positive relationship between liver fibrosis and SFA (measured in liver-PL) and a negative relationship between liver fibrosis and PUFA (in liver-PL), MUFA (in liver-TAG), and MUFA (in plasma-TAG). In particular, behenic acid (in liver-PL) was positively related, whereas EPA (in liver-PL), oleic acid (in liver-TAG), and combined oleic and vaccenic acid (in liver-TAG) were negatively associated with liver fibrosis. Certain plasma FAs mirrored their associations in the liver, such as oleic acid (in plasma-TAG) and a combination of oleic and vaccenic acid (in plasma-TAG). However, plasma (in PL) showed an opposite relationship with liver fibrosis compared to liver behenic acid (in PL). No significant associations were observed between liver fibrosis and plasma PUFA (in PL) or plasma SFA (in PL) [102]. Interestingly, supplementing PUFA in the mfat-1 (an enzyme that converts n-6 to n-3 PUFAs)-overexpressing transgenic mice model significantly prevented carbon tetrachloride-4 (CCl4)-induced liver fibrosis and reduced in AST and ALT compared to wild-type mice [103]. Similarly, in rats, PUFA supplementation showed not only a significant reduction in fibrosis but also a reversal in CCL4-induced cirrhosis. These rats also showed faster regeneration of the liver post-hepatectomy in addition to decreased IL-6 (pro-inflammatory cytokine) and increased IL-10 (anti-inflammatory cytokine) [104].

At the molecular level, when the FASN and SCD expression at the level of mRNA and protein were considered, a concurrent increase was observed [105], suggesting that once the FAs are made via DNL, these FAs are desaturated to MUFA, resulting in a strong correlation between DNL (percent) and MUFA (percent of total FAs) but not between percent DNL and percent SFA [106]. Similarly, Knebel et al. found a simultaneous increase in DNL (measured indirectly using multiple DNL calculators) and percent MUFA in C57BL6 mice. In a study conducted by Shan et al., transgenic mice overexpressing mfat-1 exhibited a reduction in CCL-4-induced increase in fibrosis-related proteins like the mechanistic target of rapamycin (mTOR) and B-cell lymphoma 2 (Bcl-2)/Bcl-2-associated X protein (Bax) [103]. In another study, N-3 PUFA supplementation suppressed CCL4-induced increased mRNA expression of cirrhotic genes, i.e., transforming growth factor-beta (TGF-β) and upregulating the expression of matrix metalloproteinase-9 (MMP-9) and matrix metalloproteinase-1 (TIMP-1) in the liver [104]. Lastly, individuals with elevated IHTG, in contrast to those with low IHTG, demonstrated a higher 16:1n7 FA mole percentage in both VLDL fraction and NEFA, and only 18:1n7 (MUFA) in NEFA [84], indicating a clear relationship between DNL and MUFA. In a study by Roumans et al. [107], a positive correlation was found between the percentage of SFA and DNL, while a negative correlation was observed between the percentage of MUFA and DNL. These findings highlight the need for further research to elucidate the precise roles of MUFA and PUFA in the pathophysiology of MASLD.

### 3.2. Triacylglycerol and Diacylglycerol

**Figure 2:**
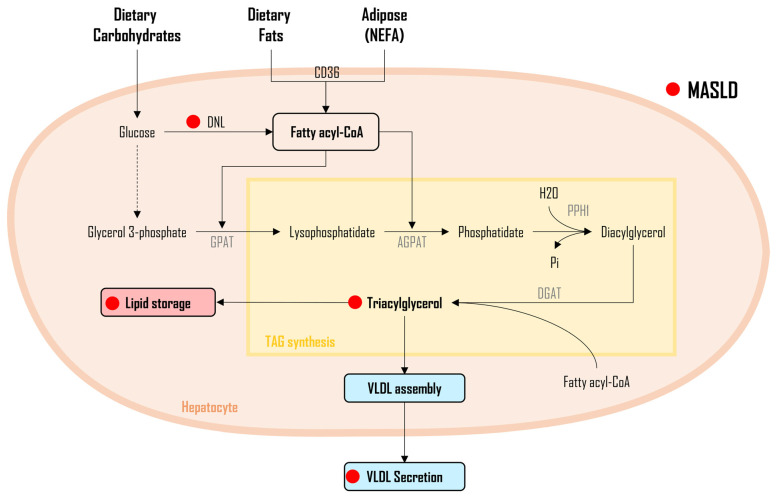
Triacylglycerol and diacylglycerol synthesis in MASLD. Legend: This figure illustrates the general metabolism of triacylglycerol (TAG) and diacylglycerol (DAG) synthesis. Three distinct sources of fatty acids (FAs) undergo enzymatic conversions, leading to the formation of fatty acyl-CoA. Notably, in MASLD, elevated DAG levels contribute to lipotoxicity, exacerbating fat accumulation, oxidative stress, inflammation, and insulin resistance. Mechanistically, DAG inhibits insulin receptor tyrosine kinase via PKC-ε activation, underscoring its significance in MASLD pathogenesis. Increased triacylglycerol (TAG) levels signify increased hepatic TAG accumulation, marking a crucial stage in MASLD progression. The red dot (●) represents changes altered during MASLD pathogenesis. Figure abbreviations: AGPAT—1-acylglycerol-3-phosphate-O-acyltransferase; CD36—cluster of differentiation 36; CE—cholesterol ester; DGAT—diacylglycerol acyltransferase enzyme; DNL—de novo lipogenesis; FA—fatty acids; GPAT—glycerol-3-phosphate; Pi—phosphate; NEFA—nonesterified fatty acids; PDAT—Phospholipid: diacylglycerol acyltransferase; PPH-1—phosphohydrolase–1; TAG—triacylglycerols; and VLDL—very-low-density lipoprotein.

As illustrated in Figure 2, three distinct sources of FAs DNL from dietary carbohydrates, non-esterified FAs (NEFA) released from adipose tissue, and FAs obtained from the diet, undergo a sequence of enzymatic conversions, resulting in the formation of fatty acyl-CoA molecules. Subsequently, fatty acyl-CoA undergoes a series of enzymatic reactions, with glycerol-3-phosphate acyltransferase (GPAT) facilitating their conjugation to glycerol-3-phosphate, leading to the synthesis of lysophosphatidate. Next, another fatty acyl-CoA is incorporated into the process through the enzymatic activity of 1-acylglycerol-3-phosphate-O-acyltransferase (AGPAT), resulting in the formation of phosphatidate. The generated phosphatidate molecules, in turn, undergo enzymatic modification via phosphohydrolase-1 (PPH-1), resulting in the production of diglycerides (DAG). It is noteworthy that DAGs can also be derived from monoglycerides (MAG), with monoacylglycerol acyltransferase (MGAT) playing a pivotal role, although this specific pathway is not depicted in Figure 2. Ultimately, the DAG is converted into triglycerides (TAG) through the enzymatic incorporation of a fatty acyl-CoA, mediated by the diacylglycerol acyltransferase enzyme (DGAT). The TAGs that have been newly synthesized undergo subsequent pathways, wherein they may either be integrated into lipoproteins, released into circulation, utilized as energy, or stored within tissues for future metabolic requirements [108]. Elevated triglyceride levels, a common trait observed in individuals with MASLD and MASH [4,109,110,111,112], function as an indicator of increased hepatic TAG accumulation, signifying the primary stage in the progression of MASLD [112,113,114,115].

In addition to TAG, the intermediate product [60,116] DAG has been identified as a contributing factor to MASLD development and progression primarily due to its lipotoxic properties [117]. Additionally, increased levels of hepatic DAG have been associated with key characteristics of MASLD i.e., fat accumulation, oxidative stress, inflammation, and insulin resistance [117]. At the molecular level, DAG has been shown to inhibit the tyrosine kinase activity of the insulin receptor [118] via activation of protein kinase C-ε (PKC-ε) [119] in the liver [60,120], which, at the intracellular level, impairs the downstream signaling cascade of insulin, resulting in insulin resistance [121]. These effects of DAG were reversed in rats with knockdown of PKC-ε expression [122]. Furthermore, when the gene encoding PKC-ε (Prkce) was deleted in mice, high-fat feeding failed to induce insulin resistance despite an increase in liver-TAG contents (likely due to lipid partitioning that promoted FA esterification in hepatocytes) [123]. Lastly, the administration of a ketogenic diet in rats also resulted in reduced levels of DGA and PKC-ε and prevented hepatic steatosis and insulin resistance development [124]. Thus, inhibiting DAGs appears to be a promising mechanism. As a result, certain pharmaceutical companies are presently engaged in the development and experimentation of a pharmaceutical agent designed to inhibit DGAT enzymes, aiming to reduce the synthesis of TAGs and ultimately decrease IHTG levels in MASH/MASLD patients [125].

### 3.3. Cholesterol

**Figure 3:**
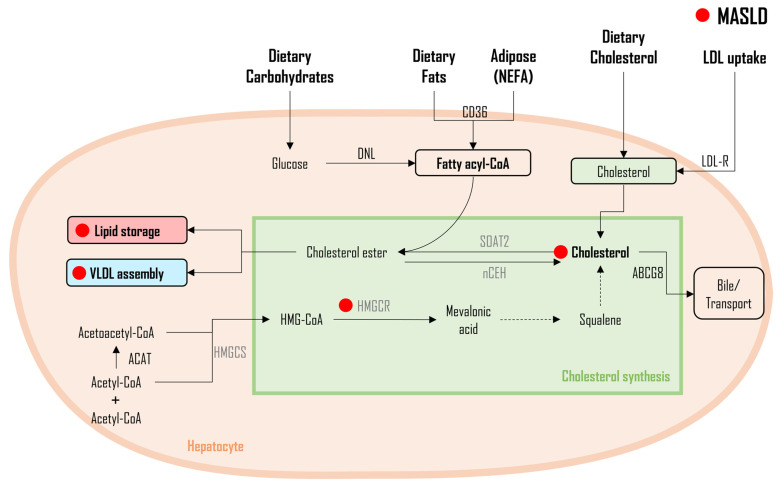
Cholesterol synthesis and implications in MASLD. Legend: This figure outlines cholesterol synthesis, with acetyl-CoA acetyltransferases initiating the pathway and HMG-CoA reductase progressing to cholesterol. In MASLD, elevated total cholesterol and LDLc, along with reduced HDLc, are observed. SREBP-2, a master regulator, shows increased expression, contributing to heightened cholesterol synthesis. Enhanced HMGCR activity in MASLD and MASH exacerbates cholesterol production, impacting liver function. Altered cholesterol metabolism plays a crucial role in MASLD progression, with potential implications for inflammation and fibrosis within liver cells. The red dot (●) represents changes altered during MASLD pathogenesis. Figure abbreviations: ABCG8—ATP-binding cassette sub-family G member 8; ACAT—acetyl-CoA acetyltransferases; DNL—de novo lipogenesis; HMGCR—β-Hydroxy β-methylglutaryl-CoA reductase; HMGCS—β-Hydroxy β-methylglutaryl-CoA synthase; LDL-R—low-density lipoprotein receptor; NCEH1—neutral cholesterol ester hydrolase 1; NEFA—nonesterified fatty acids; and SOAT2—sterol O-acyltransferase 2.

Depicted in Figure 3 is the synthesis of cholesterol. Catalyzed by acetyl-CoA acetyltransferases (ACAT), two molecules of acetyl-CoA transform into acetoacetyl-CoA. Later, another molecule of acetyl-CoA is further added to acetoacetyl-CoA to form a molecule that plays a central role in the cholesterol synthesis pathway known as β-hydroxy β-methylglutaryl-CoA (HMG-CoA). In the presence of the HMG-CoA reductase enzyme (HMGCR), HMG-CoA undergoes a series of reactions to eventually produce mevalonic acid. Mevalonic acid is then converted into squalene and eventually to cholesterol [23,126]. The metabolic fate of cholesterol includes the synthesis of bile acid, which is transported through an ATP-binding cassette sub-family G member 5/8 (ABCG5/8). Additionally, cholesterol can be esterified to produce CE in the presence of an enzyme called acyl-coenzyme A: cholesterol acyltransferase 2 (ACAT2), also termed SOAT2. Lastly, cholesterols can also be incorporated into very-low-density lipoprotein (VLDL) particles for secretion or can be stored in the form of a lipid droplet for later utilization [126]. In the context of MASLD pathophysiology, numerous studies have reported elevated levels of total cholesterol and low-density lipoprotein cholesterol (LDLc) concentrations in plasma. Conversely, reduced concentrations of high-density lipoprotein cholesterol (HDLc) have been observed in patients with MASLD when compared to those without MASLD [4,109,110,111,112,127]. One of the key mechanisms underlying these alterations in cholesterol concentrations implicates the involvement of the SREBP-2 gene, a master regulator of the cholesterol pathway, which activates HMGCR [126]. In a study conducted in patients diagnosed with MASH and those diagnosed with simple steatosis only, a significantly higher expression of SREBP-2 was observed in patients with MASH and fibrosis compared to those with or without steatosis [128,129]. In *Alms1* mutant (*foz*/*foz)* mice treated with a high-fat diet, there was a marked increase in the overexpression of SREBP-2 compared to wild-type mice [130]. These mice exhibited elevated concentrations of free cholesterol (FC), which has been attributed as a potential causative factor in the pathophysiological progression of steatosis to MASH [130]. These results primarily stemmed from an elevated LDL-receptor (LDL-R)-mediated uptake of cholesterol, the reduced involvement of pathways involved in the synthesis of bile, and decreased secretion from the liver [130,131]. Furthermore, these mice demonstrated increased cholesterol esterification, a phenomenon supported by heightened SOAT2 activity. Min et al. analyzed liver biopsies obtained from human subjects and demonstrated increased FC levels in patients with both MASLD and MASH relative to those without any liver disease [132]. The elevated levels of FC in patients with MASLD/MASH were attributed to enhanced HMGCR activity resulting in increased synthesis, as opposed to alterations in the uptake of cholesterols through LDL-R or reductions in the synthesis/export of bile [132]. In the same study [132], individuals who were prescribed statins displayed noticeably reduced SREBP2 and HMGCR mRNA expression in their liver samples in comparison to those who were not taking statins. In a study involving diabetic and obese mice, the administration of atorvastatin and ezetimibe, both of which target cholesterol production and uptake by HMGCR inhibition and the Niemann–Pick C1-like 1 (NPC1L1) proteins, led to the reversal of fibrotic MASH [133]. Nonetheless, bile acid metabolism also plays a critical role in MASLD pathogenesis. The disrupted intricate balance of bile acid synthesis (via cholesterol 7α-hydroxylase (CYP7A1)) transport (ABCG5/8/B11 and SLC10A1) and signaling pathways (primarily regulated by the farnesoid X receptor (FXR) and the liver X receptor (LXR)—key nuclear receptors that govern bile acid metabolism) contributes to the progression of hepatic steatosis and inflammation [134,135,136]. When steroidogenic acute regulatory protein (StarD1) was overexpressed in western-diet-fed mice, bile acid synthesis increased, resulting in reduced hepatic lipid content (TAG, total cholesterol, and free FAs) [137]. Despite a reduction in hepatic total cholesterol, oxysterol 7α-hydroxylase (CYP7B1, a gene responsible for oxysterols to bile acids) was significantly inhibited by increased StarD1 activity. Interestingly, CYP7B1 is also reported to be reduced in the presence of insulin resistance (a characteristic common in the early stages of MASLD), resulting in the increased accumulation of hepatic sterols, eventually leading to increased inflammation [137]. Therefore, previous research strongly substantiates the role of the altered metabolism of cholesterols in MASLD [138,139], and its more severe manifestation, MASH. Cholesterol plays a contributing role in worsening inflammation and fibrosis within liver cells [140]. Consequently, the management of cholesterol concentrations has been a common recommendation as a therapeutic approach for MASLD management [141]. 

### 3.4. Ceramides

**Figure 4:**
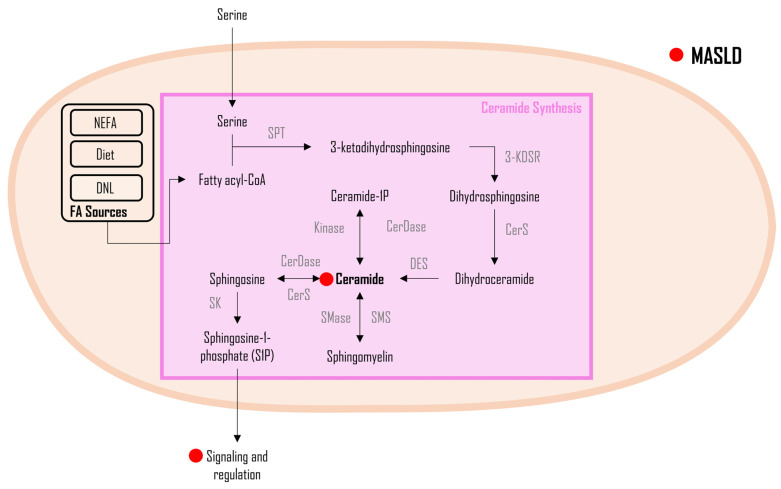
Ceramide metabolism in MASLD. Legend: This figure outlines ceramide synthesis and impacts, emphasizing crucial roles in cell membrane integrity, skin barrier function, apoptosis, and lipid metabolism. Elevated ceramide levels in metabolic disorders, cardiovascular diseases, and MASLD are linked to increased lipid peroxidation and reduced mitochondrial respiration in MASH. Ceramides contribute significantly to hepatic steatosis and insulin resistance by activating PKCζ and PP2A, impairing AKT translocation, suppressing β-oxidation, and activating the NLRP3 inflammasome. The red dot (●) represents changes altered during MASLD pathogenesis. Figure abbreviations: 3-KDSR—3-ketodihydrosphinganine reductase; C1P—ceramide-1-phosphate; Cer—ceramides; CerDase—ceramidase; CerS—ceramide synthase; DES—dihydroceramide desaturase; S1P—sphingosine-1-phosphate; SK—sphingosine kinase; SMase—sphingomyelinase; SMS—sphingomyelin synthase; and SPT—serine palmitoyltransferase.

Ceramides, a lipid class within the sphingolipid family, have recently garnered attention due to their lipotoxic properties and their integral role in cell membrane composition [117,142]. As shown in Figure 4, ceramides, vital lipid molecules for cell membrane integrity, are synthesized in the endoplasmic reticulum and Golgi apparatus [143]. The process involves condensing a long-chain fatty acid with serine, catalyzed by serine palmitoyltransferase (SPT) to form 3-ketodihydrosphinganine. Subsequent enzymatic steps lead to dihydroceramide, which is converted into ceramide through desaturation by dihydroceramide desaturase [144]. Ceramides are involved in skin barrier function, apoptosis regulation, and lipid metabolism [145,146]. Ceramides produce metabolic byproducts, including sphingosine, which regulates cell growth and apoptosis; ceramide-1-phosphate (C1P) involved in cell signaling and inflammation; sphingomyelin, which is crucial for cell membrane integrity; and sphingosine-1-phosphate (S1P), a signaling molecule influencing cell migration and immune responses [143].

Elevated cellular ceramide concentrations have been previously linked to metabolic syndrome, cardiovascular diseases, and MASLD, with specific ceramide ratios (such as d18:1/16:0)/(d18:1/24:0) serving as significant predictors of cardiovascular events and mortality [117,147,148,149,150,151,152,153]. Notably, a study in patients with MASH and insulin resistance has demonstrated that increased hepatic ceramide levels are linked to higher lipid peroxidation and reduced mitochondrial respiration [154]. Hepatic diacylglycerol (DAG) levels have also been correlated with increased insulin resistance, fat accumulation, oxidative stress, and inflammation—factors that are prevalent in MASLD [117,155,156]. Ceramides also substantially contribute to the augmentation of hepatic steatosis and insulin resistance in obesity by activating PKCζ and protein phosphatase 2A (PP2A), leading to impaired AKT translocation and dephosphorylation of AKT [117]. Ceramides inhibit β-oxidation and activate the nucleotide-binding domain, leucine-rich-containing family, pyrin domain-containing 3 (NLRP3) inflammasome [157], further contributing to hepatic steatosis and insulin resistance.

Fecal calprotectin is a protein that indicates the migration of neutrophils, which commonly occurs during inflammation of the gut. In a study conducted by Feysa et al., fecal calprotectin was significantly higher in the steatosis grade 2 and 3 groups compared to that in the grade 1 group, suggesting a correlation between fecal calprotectin and steatosis grade [158]. These findings were corroborated by Demirbaş et al. who also reported significantly higher fecal calprotectin levels in obese and MASLD patients compared to non-obese healthy individuals [159]. Further, FC levels reported in cirrhotic patients were much higher than in the control group [160,161] suggesting a direct relationship between fecal calprotectin and MASLD progression.

## 4. Lipid oxidation in MASLD

### FA Oxidation

**Figure 5:**
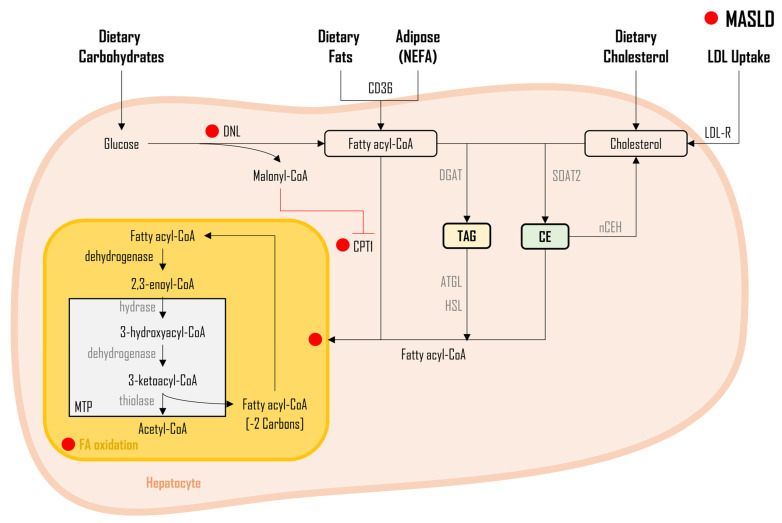
Fatty acid oxidation in MASLD. Legend: Illustration of diverse fatty acid sources contributing to cellular oxidation in non-alcoholic fatty liver disease (MASLD). Carnitine palmitoyltransferase 1 (CPT1), a pivotal outer-membrane mitochondrial enzyme, facilitates fatty acid uptake and oxidation through the ß-oxidation pathway. Malonyl-CoA, an intermediate in de novo lipogenesis (DNL), inhibits CPT1, preventing the entry of fatty acids from plasma non-esterified fatty acids (NEFA), diet, triglyceride (TAG) catabolism, and cholesterol ester (CE) hydrolysis into mitochondria for oxidation. This dysregulation results in hepatic lipid accumulation, fostering the progression of liver steatosis and metabolic dysfunction. The red dot (●) represents changes altered during MASLD pathogenesis. *Figure abbreviations:* 2C—two carbons; ATGL—Adipose triglyceride lipase; CD36—cluster of differentiation 36; CE—cholesterol esters; CPT1—carnitine palmitoyltransferase 1; DGAT—diacylglycerol acyltransferase enzyme; DNL—de novo lipogenesis; FA—fatty acids; HSL—hormone-sensitive lipase; LDL-R—low-density lipoprotein receptor; MTP—mitochondrial trifunctional protein; NCEH—neutral cholesterol ester hydrolase; NEFA—non-esterified fatty acids; SOAT2—sterol O-acyltransferase 2; and TAG—triacylglycerols.

As illustrated in Figure 5, besides the FAs originating from DNL, dietary intake, and NEFA, additional sources of FAs stem from TAG catabolism and CE hydrolysis. These FAs are subject to cellular oxidation to meet energy demands [162]. Carnitine palmitoyltransferase 1 (CPT1) plays a pivotal role as an outer-membrane mitochondrial enzyme, facilitating FA uptake and their subsequent oxidation through the ß-oxidation pathway [163,164,165,166]. Typically, each FA undergoes oxidation, hydration, oxidation, and thyolisis, respectively—four primary steps of ß-oxidation, with two carbons being eliminated from FA during each cycle [167,168]. The final three steps occur within a protein assembly known as mitochondrial trifunctional protein (MTP) [169,170,171]. For example, for a common 16 carbon-containing FA like palmitate to achieve complete oxidation, palmitate must undergo a total of eight cycles, resulting in the production of eight acetyl-CoA molecules. Previous literature discussing FA oxidation’s role in MASLD pathophysiology reported conflicting outcomes [112,172,173,174,175,176,177,178,179,180,181,182,183]. Nevertheless, most of these investigations assessed ß-hydroxybutyrate as a surrogate measure of ß-oxidation [112,172,173,174,178,182]. Kotronen et al. found no differences in ß-hydroxybutyrate levels between MASLD and non-MASLD obese subjects [182]. On the contrary, several other studies noted a decrease in fatty acid oxidation in MASLD. This reduction was assessed through direct measurement of CPT1 or PPAR-α, as well as indicators such as breath ^13^CO_2_, plasma ^13^C-NMR, or ß-hydroxybutyrate [176,177,178,179,180,181,184,185]. Croci et al. [178] observed a reduction in ß-oxidation, evidenced by lower concentrations of plasma ß-hydroxybutyrate in patients with MASLD when compared to their healthy counterparts. In a separate investigation by Fletcher et al., despite significantly lower rates of ketone synthesis observed in MASLD patients, their ß-oxidation (assessed via ^13^C-NMR) revealed no significant differences compared to healthy individuals [179]. This suggests that using ketone synthesis as a surrogate for FA oxidation may not be a reliable marker. In an investigation, Naguib et al. assessed oxidation of FAs through breath measurements, and reported a decrease in the oxidation of dietary FAs in MASLD patients in comparison to those without MASLD [184]. Fletcher et al. proposed that NEFA may not be the only source of lipid-driving lipid oxidation in patients with MASLD, as ß-oxidation showed no significant differences despite reduced NEFA levels [179]. Although the MTP protein was demonstrated to regulate FA oxidation in MASLD [186], most studies associated decreased fatty acid oxidation with an elevated DNL [187,188,189]. This occurs because malonyl-CoA, a DNL pathway intermediate, inhibits CPT1 activity [187,188,189]. This inhibition prevents the entry of FAs sourced, not only from the pools of plasma NEFA, but also from the diet, into the mitochondria for oxidation [160,177,178,190] (Figure 5). In a more recent understanding of complete mitochondrial FAO (i.e., oxidation of FAs to CO_2_) and its rate-limiting enzyme (β-hydroxyacyl-CoA dehydrogenase) activity, MASH patients exhibited significantly lower complete whole liver FAO, mitochondrial FAO, and β-hydroxyacyl-CoA dehydrogenase activity. Moreover, when patients were grouped based on the presence or absence of fibrosis, only complete mitochondrial FAO was significantly lower, along with increased oxidative stress and decreased mitochondrial biogenesis. These data suggest that mitochondrial FAO plays a crucial role in the development and progression of MASLD to its more severe forms, MASH, and fibrosis [31,185]. Lastly, since acylcarnitine is an intermediate molecule produced during FAO, it has been used as a marker for incomplete FA oxidation. In a study conducted by Enooku et al. [191], long-chain actylcarnitine species increased gradually and significantly in fibrosis and HCC in biopsy-proven MASLD patients’ serum samples. These data suggest that FA oxidation decreases as the disease progresses from MASLD to its more severe forms, i.e., MASH, fibrosis, and HCC.

## 5. Lipid Flux and Storage in MASLD

**Figure 6:**
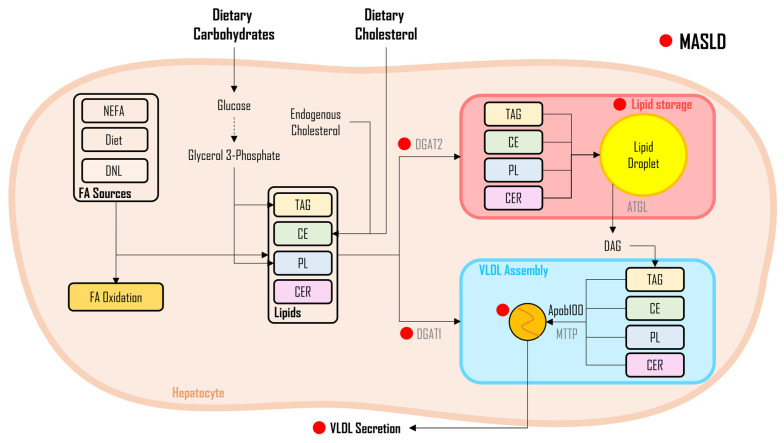
Lipid flux in MASLD. Legend: This figure illustrates the intricate fate of fatty acids (FAs). FAs from de novo lipogenesis (DNL), non-esterified FAs (NEFA), and dietary fats undergo oxidation for energy or are utilized in lipid biosynthesis (triglycerides [TAG], ceramides [CER], and phospholipids [PL]). While DGAT1 activation drives FA incorporation into lipoprotein particles (VLDL) for circulation, DGAT2 activation leads to storage in hepatic lipid droplets. Insulin resistance in MASLD diminishes insulin-induced suppression of VLDL synthesis, contributing to elevated VLDL secretion and subsequent lipid dysregulation. The red dot (●) represents changes altered during MASLD pathogenesis. Figure abbreviations: APOB100—apolipoprotein B100; ATGL—adipose triglyceride lipase; CE—cholesterol esters; DAG—diacylglycerols; DGAT—diacylglycerol acyltransferase enzyme; DNL—de novo lipogenesis; FA—fatty acids; MTTP—microsomal triglyceride transfer protein; NEFA—nonesterified fatty acids; PL—phospholipids; TAG—triacylglycerols; and VLDL—very-low-density lipoprotein.

As illustrated in Figure 6, FAs appearing in the circulation stemming from all three sources (i.e., DNL, adipose tissue, referred to as NEFA, and dietary fats) primarily undergo two metabolic fates. They are either subjected to oxidation for energy production (to be elaborated upon later) or used in the biosynthesis of lipids (i.e., TAG, CER, and PL) using glycerol 3-phosphate or esterification of cholesterols utilizing cholesterol derived from exogenous or endogenous sources [40]. Depending on the body’s needs or the presence of pathological conditions, synthesized lipids are either secreted into circulation through the VLDL particles [114,126,139] or they are stored as lipid droplets in the liver. While the former mechanism appears to be predominantly activated by DGAT 1, i.e., the incorporation of fats into the lipoprotein particles in conjunction with apoB100 [114,192,193]; the later fate (storage via incorporation of fats into lipid droplets) appears to be driven by the activation of DGAT2 and is subsequently employed by lipolysis. In healthy males, the in vivo administration of insulin during an euglycemic hyperinsulinemic clamp resulted in decreased NEFA concentrations and reduced synthesis and secretion of VLDL [194]. Nevertheless, in individuals with insulin resistance, these insulin-induced suppressive effects appeared to be diminished, ultimately resulting in increased VLDL synthesis and secretion [195]. Hamsters with insulin resistance exhibited elevated apoB100 production, primarily due to microsomal triglyceride transfer protein (MTTP) overexpression, increased neutral lipids availability in the liver, and decreased apoB100 degradation. Consequently, these factors contributed to the increased overproduction, assembly, and secretion of VLDL particles [196,197]. Debate surrounds the causative factor behind fatty liver development, with two potential mechanisms being considered. One hypothesis suggests that fatty liver arises from the suppression of VLDL-TAG secretion. Alternatively, it may be attributed to an increased VLDL-TAG secretion that, despite being elevated, remains insufficient to stimulate lipid synthesis adequately in the liver. A correlation between the percentage of VLDL-TAG accounted for and the percentage of IHTG accounted for in patients with MASLD was observed [75]. Specifically, following a five-day regimen of infusing and feeding isotopically labeled FAs, the individuals in whom the liver exhibited the greatest degree of labeling were the same individuals whose VLDL particles were enriched with the most distinct labeling. The contributions, stemming from all three sources of FAs, were consistent between the liver and VLDL-TAG. Furthermore, individuals with MASLD exhibited compromised insulin-induced inhibition of VLDL kinetics, leading to increased VLDL concentrations as well as apoB100 in those with elevated IHTG content, in contrast to those with low IHTG levels [76,198]. Additionally, it is worth noting that individuals with MASLD who exhibit high levels of IHTG were unable to inhibit the release of VLDL particles and apoB100 protein effectively during a clamp, indicative of insulin resistance in the liver [198]. These manifestations primarily arise from elevated TAG synthesis originating from FAs derived from non-systemic sources like DNL [76,115]. Nonetheless, in an investigation involving morbidly obese individuals, VLDL kinetics were assessed in cohorts with varying levels of IHTG. The study’s results suggest that the elevated IHTG levels were more likely associated with a decline in VLDL secretion instead of an increase in plasma NEFA uptake in the liver [199]. Interestingly, the secretion rates of the VLDL particles remained greater than those observed in previous studies involving obese individuals [200]. Consistent with these findings, the aforementioned results were corroborated by another study indicating reduced rates of VLDL synthesis and secretion in individuals with high liver fat relative to those with low liver fat [32]. The reduced VLDL secretion observed in individuals with MASH appears to be due to impaired synthesis rates of VLDL particles and a reduction in apoB100 and MTTP (a protein essential for integrating TAG into VLDL particles [201]) mRNA expression levels [32]. In a study led by Charlton et al., the assessment of the rate of synthesis of apoB100 unveiled lower rates in individuals with MASH compared to both obese and lean counterparts, indicating a potential implication of apoB100 synthesis in MASH development [202]. On the contrary, Smith et al. conducted a study that documented increased VLDL secretion in obese men without MASLD [203]. Interestingly, in biopsy-proven MASLD patients with fibrosis, VLDL mass, VLDL-TAG content, and a proportion of large VLDL subfractions were significantly reduced [204]. Collectively, these findings indicate that VLDL flux (both the synthesis and secretion of the particles) likely plays a pivotal role in the development and progression of MASLD and its severe forms (MASH and fibrosis). Nevertheless, it is important to underscore that the VLDL secretion observed in the pathogenesis of MASLD and MASH could be inadequate to sufficiently match with significantly higher rates of TAG synthesis recorded in patients with MASLD/MASH.

## 6. Interplay of Cholesterol Metabolism and DNL in MASLD

Regarding the correlation between cholesterol levels and the progression of MASLD, elevated lipid levels are frequently observed in MASLD patients [4,109,110,111,112]. In a lipidomic analysis of liver tissue obtained from healthy individuals, MASLD patients, and MASH patients, the concentrations of liver-CE were not significantly different between all groups. Regardless, in another study, FC concentrations were reported to be higher in patients with MASH only [100]. However, for total cholesterol levels, both MASLD and MASH patients exhibited higher concentrations with no differences in HDLc and LDLc, suggesting greater cholesterol content in VLDL and IDL remnant particles [100]. In another study, total cholesterol levels exhibited no distinctions; nonetheless, LDLc was markedly elevated in both groups of patients (i.e., MASLD and MASH) when compared to their healthy counterparts. Simultaneously, HDLc was lower only in the MASH group [132]. When gene analysis was performed, genes involved with cholesterol synthesis (i.e., HMGCR increased), clearance (LDL-R reduced), and those involved in the synthesis of bile (CYP 7A1/27A reduced) or transport (ABCG1/8 reduced) were altered, which may have resulted in higher FC in MASH patients. Importantly, in this study, a noteworthy linear association was identified between HMGCR expression and the MASLD activity score (NAS). This implies an increase in cholesterol synthesis as MASLD advances into more severe stages like MASH, fibrosis, and cirrhosis. Whether this relationship exists with total FC remains unexplored.

SREBP2 serves as a vital regulator in cholesterol pathways, essential for generating an endogenous sterol ligand activating SREBP1c [24]. Intriguingly, the concomitant increase in TAG and cholesterols observed in patients with MASH, along with overexpression of the key master regulator, SREBP-2, clearly indicates a potential interplay between both the pathways of FA synthesis (i.e., the DNL) and pathways involving the synthesis of cholesterols [24,129]. Acetoacetyl-CoA has been proposed as a potential link connecting these two pathways [205]. This occurs because, within the DNL pathway, acetyl-CoA combines with malonyl-CoA, resulting in the formation of an acetoacetyl-CoA molecule (also referred to as ß-ketoacyl-ACP). Conversely, in the cholesterol pathway, two acetyl-CoA molecules give rise to acetoacetyl-CoA. Therefore, it is likely that the acetoacetyl-CoA molecule generated during lipogenesis by the FASN enzyme could potentially be routed toward the cholesterol synthesis pathway [205]. Additionally, the positive associations reported between the DNL percent and plasma total cholesterol and plasma LDLc provide additional evidence for this notion [206]. Interestingly, the data reported from both animal models and human studies showed that the pharmacological inhibition of DNL significantly reduced all forms of cholesterols (total, HDL, and LDL), which further supports the concept of the interplay between DNL and cholesterol pathways [46,48,50,90,91,92,93]. Considering this data, the severity of MASLD may correspondingly elevate both DNL and cholesterol synthesis pathways.

## 7. Conclusions

In summary, the intricate interplay of lipid metabolic pathways elucidates the multifaceted progression of MASLD and its evolution into MASH. The synthesis of TAG and DAG, coupled with the integration of cholesterol, ceramides, and lipid oxidation collectively unveils the complex pathophysiology underlying hepatic dysfunction. Elevated TAGs, in correlation with the cholesterol dynamics governed by SREBP-2, and the lipotoxic potential of ceramides emerge as pivotal players in the pathogenesis of MASLD disease. The coordination of VLDL kinetics, a dynamic interplay of synthesis and secretion, highlights its central role in hepatic lipid homeostasis. Amidst these molecular complexities, targeted interventions directed at key enzymatic mediators (particularly those involved in DNL and DAG/TAG metabolism) present promising avenues for therapeutic investigation in the realm of MASLD and MASH. This review serves as a steppingstone for future research, sparking hypotheses and guiding the way for more targeted investigations in the complex field of hepatic lipid metabolism, ultimately aiming to enhance clinical strategies for managing and eventually treating MASLD and MASH.
